# Current Evidence and Future Directions of Berberine Intervention in Depression

**DOI:** 10.3389/fphar.2022.824420

**Published:** 2022-05-23

**Authors:** Wen-Qian Zhu, Hui-Ying Wu, Zhi-Hui Sun, Yi Guo, Tong-Tong Ge, Bing-Jin Li, Xin Li, Ran-Ji Cui

**Affiliations:** ^1^ Jilin Provincial Key Laboratory on Molecular and Chemical Genetic, Second Hospital of Jilin University, Changchun, China; ^2^ Department of Pharmacy, The Eastern Division of First Hospital of Jilin University, Changchun, China; ^3^ Department of Breast Surgery, The Affiliated Hospital Changchun University of Chinese Medicine, Changchun, China

**Keywords:** berberine, bioamine, depression, natural active ingredients, neuroplasticity

## Abstract

A major type of serious mood disorder, depression is currently a widespread and easily overlooked psychological illness. With the low side effects of natural products in the treatment of diseases becoming the pursuit of new antidepressants, natural Chinese medicine products have been paid more and more attention for their unique efficacy in improving depression. In a view from the current study, the positive antidepressant effects of berberine are encouraging. There is a lot of work that needs to be done to accurately elucidate the efficacy and mechanism of berberine in depression. In this review, the relevant literature reports on the treatment of depression and anxiety by berberine are updated, and the potential pharmacological mechanism of berberine in relieving depression has also been discussed.

## Introduction

Depressive disorder is a heterogeneous disease based on symptoms forming a syndrome and causing bodily dysfunction, which has the characteristics of multiple phenotypes, biological and psychological susceptibilities, and indirect incidence (Kim 2021). The cardinal symptoms of patient include emotional instability, anhedonia, poor concentration, and tendency to commit suicide (Yang et al., 2015). With the increasing number of patients, depression has become a serious illness that threatens human health, and increases burden on families and society. A lot of research reports have made some breakthroughs in elucidating the underlying pathogenesis of depression, mainly including the widely accepted monoamine hypothesis, hypothalamic–pituitary–adrenal axis disorder hypothesis, neuroinflammation hypothesis, and neuroplasticity and neurogenesis hypothesis (Malhi and Mann 2018). These hypotheses show that depression is a rather complex medical disease caused by a combination of genetic and environmental factors, involving a variety of neurobiological substrates, brain regions, and circuits (Wang et al., 2019). Although multiple antidepressants are available, most patients do not respond with the desired effect to these treatments and even induced paradoxical effects, such as deterioration of symptoms and withdrawal symptoms (Chouinard and Chouinard 2015). In the search for better antidepressants, the lower side effects of drugs have become a pursuit of new antidepressants. Therefore, natural Chinese medicine products have been widely a concern by medical workers for their unique efficacy in improving depression. In particular, the research of safe and effective antidepressants in traditional herbal medicine has become a hot spot in the current medical field.

**FIGURE 1:**
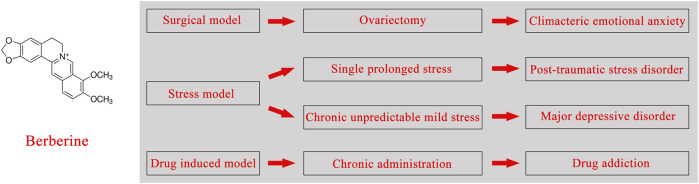
Chemical structural formula of berberine, associated depression models, and clinical diseases.

The naturally effective ingredients tested in most studies were selected based on their multiple pharmacological actions, medicinal value, or as antidepressants. Berberine, an isoquinoline alkaloid, was extracted from the roots and aboveground parts of plants (Table 1), which are readily available from Berberidaceae, Papaveraceae, Menispermaceae, Ranunculaceae, and other botanical families (Kulkarni and Dhir 2010; Liu et al., 2016). Clinical studies of berberine have revealed multiple therapeutic actions, including anti-inflammatory, anticancer, relieving depression, hypolipidemic, and hypoglycemic actions, which indicate that berberine is a drug with multispectral activities (Mujtaba et al., 2021). Therefore, it is also a noteworthy candidate drug for the treatment of depression, which has been studied in several animal models of depression (Figure 1). The purpose of this review was to investigate the mechanism of berberine and its traditional Chinese medicine prescription in the treatment of depression in recent literature reports, combined with the pathogenesis of depression. Meanwhile, we sought to present a summary of the current problems and feasible solutions of berberine in the clinical treatment of depression.

**TABLE 1:** Representative examples of potential plant sources of berberine with medicinal value.

No	Botanical name	Part used	Modal	Pharmacological properties	Reference
1	*Argemone mexicana* L	Leaves	Bacterial species (G+/G−)	Antibacterial activity	More et al. (2017)
2	*Berberis amurensis*	Root	Ear swelling mice	Anti-inflammatory and analgesic	Liao et al. (2021)
3	*Berberis aquifolium*	Root	HPV-positive and HPV-negative CaCx cell lines	Anticancer	Singh et al. (2021)
4	*Berberis aristate* L	Mixture of plant extracts	C57BL/6J mice/metabolic syndrome patients	Amelioration of metabolic parameters and of hepatic steatosis	Roshanravan et al., (2020) Cossiga et al. (2021)
5	*Berberis asiatica*	Air-dried root, stem	Multiple bacterial strains, fungi strains	Antibacterial activity, antifungal activity	Singh et al. (2007)
6	*Berberis chitria*	Air-dried root, stem	Multiple bacterial strains, fungi strains	Antibacterial activity, antifungal activity	Singh et al. (2007)
7	*Berberis croatica* Horvat	Root and twig	Bacterial species (G+/G−) and yeast	Antimicrobial activity	Kosalec et al. (2008)
8	*Berberis lycium* Royle	Root	HepG2 Cells	Anticancer	Mustafa et al. (2020)
9	*Berberis sibirica* Pall	Overground parts	Naive male Swiss mice	Neuroprotection	Szalak et al. (2021)
10	*Berberis thunbergii* DC.	Root	Multiple cancer cell lines	Anticancer	Suchomelova et al. (2007), Och et al. (2019)
11	*Berberis vulgaris* L	Root and twig	Bacterial species (G+/G−) and yeast	Antimicrobial activity	Kosalec et al. (2008)
12	*Coptidis rhizome* (Coptis chinensis Franch)	Dried roots	Rat/mice intestinal content	Antibacterial and antidiabetic activity	Lyu et al. (2021)
13	*Coscinium fenestratum*	Dried stems	HL-60 leukemia cells and PBMC cells	Anticancer	Tungpradit et al. (2010)
14	*Fibraurea recisa* Pierre	Fresh rattan stem	Multiple fungi strains	Antifungal activity	Rao et al. (2009)
15	*Hydrastis canadensis* L	Root and rhizomes	*Fusarium oxysporum*	Antifungal activity	Tims and Batista (2007), Douglas et al. (2010)
16	*Mahonia bealei* (Fort.) Carr	Leaves	HT-29 cells and human embryonic kidney cells	Antiproliferative activity	Hu et al. (2011)
17	*Thalictrum foliolosum* DC	Rhizomes or whole plant	β-carotene linoleate model system, goat liver, and bacterial species (G+/G−)	Antioxidant and antimicrobial activity	Pandey et al. (2018), Sharma et al. (2020)
18	*Tinospora sinensis* (Lour.) Merr	Fresh mature stems	Diabetic rat model	Antioxidant, anti-inflammatory, and antihyperglycemic activity	Banerjee et al. (2020)

Note. There are many plants potentially containing berberine, which is reflected in many folk medical records of different regions, well-documented pharmacopeia, and research reports. Among them, the plants containing berberine and used for pharmacological studies are listed in this table. Then research reports without models and therapeutic action were ignored.

Note G+, Gram-positive bacterial species; G−, Gram-negative bacterial species; HL-60 leukemia cells, acute promyelocytic leukemia; PBMC cells, peripheral blood mononuclear cells; CaCx, cancer of the cervix; HPV, human papilloma virus; HepG2 cells, hepatocellular carcinoma cells; MCF7 cell lines, human breast adenocarcinoma cell line; HT-29, human colon cancer cells;

### The effect of berberine on symptom clusters of depression

The multi-angle regulatory mechanism of berberine may confer positive therapeutic effects against depression, due to its extensive range of biological effects. Many potential factors that are well known to increase the risk of depression, including the influence of sex hormones, a dulled response to stress in the hypothalamic–pituitary–adrenal axis, and a higher tendency to introspection, might boost the chances of women suffering from depression (Riecher-Rossler 2017). Moreover, findings from a Chinese woman-based cohort study showed that symptoms of anxiety and depression are more common during and after menopause than in premenopausal women (Tang et al., 2019). Therefore, the ovariectomized (OVX) mice model was used to mimic clinical postmenopausal condition (Rana et al., 2022). Researchers have found that after gavage administration of berberine (100 mg/kg), the increased content of equol (a chemical structure similar to the hormone estrogen that binds to estrogen receptors) in the OVX rat model transplanted with fecal microbiota, enriched the taxa involved in isoflavone biotransformation and significantly reduced anxiety-like behavior. The results suggest that berberine may alleviate anxiety-like behavior in perimenopausal rat partly through the microbiota–gut–brain axis system (Fang et al., 2021). In addition, our previous study showed that ovariectomy reduced the brain-derived neurotrophic factor (BDNF) protein expression levels in the hippocampus and phosphorylated cyclase adenosine monophosphate response element-binding protein (pCREB)/CREB ratio in the frontal cortex, but this change was reversed in the short term by berberine, considering that the rapid-onset antidepressant-like behavior is mediated by the decreased phosphorylation of eukaryotic elongation factor 2 (eEF2) and the increased translation of BDNF. In this case, we suggest that berberine may produce antidepressant-like effects in OVX mice via the BDNF-CREB and eEF2 pathways (Fan et al., 2017).

Depression is closely related to a decrease in neuronal plasticity. BDNF is associated with changes in neuroplasticity in certain areas, such as the amygdala and hippocampus (Camuso et al., 2021), and is deemed to be beneficial in neurogenesis, neural survival, and growth (Shen et al., 2020). A recent study showed that berberine was involved in the regulation of depression progression through miRNA and BDNF. Berberine treatment could effectively reverse the promotion effect of overexpression of miR-34b-5p/miR-470-5p on the depressive-like behavior of chronic unpredicted mild stress (CUMS) mice and regulate the growth of hippocampal neurons by mediating the expression of BDNF(Zhan et al., 2021). Clinical studies on major depression patients showed that their hippocampus was seriously damaged, and their neurogenesis function was impaired (Park 2019; Huang et al., 2020). The above pathologic phenomenon was reversed by antidepressant treatment, suggesting that neurogenesis is associated with the altered course of depression (Zeng et al., 2022). Neurogenesis, an important focus of hippocampal structural plasticity, produces full neural function that involves the proliferation and differentiation of neural stem cells (Li et al., 2020). Berberine was able to protect C17.2 neural stem cells from the 2,2′-azobis(2-amidinopropane)dihydrochloride-induced oxidative damage; to be more specific, it lowered the cellular reactive oxygen species level in C17.2 cells via the nuclear factor erythroid 2-related factor 1/2 (NRF1/2)-NAD(P)H quinone dehydrogenase 1–heme oxygenase 1 pathway. Besides, berberine enhanced the viability of C17.2 cells by upregulating the expression of extracellular signal-related kinase (ERK) and phosphor-ERK, which contributed to the activation of the WNT/β catenin pathway in C17.2 cells to promote neuronal differentiation (Shou et al., 2019).

In addition, berberine plays an antidepressant role by regulating brain neurotransmitters, especially biogenic amines. It has high affinity for dopamine receptors (Lee et al., 2018), and regulates hypothalamic corticotrophin-releasing factor and central norepinephrine systems (Lee et al., 2012). There is evidence that berberine increases the level of dopa/dopamine in blood, in which dopa enters the brain through the circulation and is converted into dopamine, so as to improve brain function. Generally, the increase in dopamine is closely related to positive emotions (Hamon and Blier 2013). Lee et al. found that single prolonged stress induced DAergic dysfunction in rats, leading to depression-like behavior. The concentration of dopamine in the hippocampus and striatum were significantly increased after intraperitoneal injection of berberine, which may alleviate anxiety to some extent (Lee et al., 2018). A recent study showed that oral berberine could promote the production of dopa/dopamine by improving the synthetic pathway of Phe–Tyr–dopa–dopamine in the gut flora, and the active compound produced by dopa/dopamine is dihydroberberine (an intestinal metabolite). In other words, dopa/dopamine in brain tissue was increased by dihydroberberine, but not berberine (Wang et al., 2021). These advances may lead to a deeper understanding of the role of berberine in regulating biogenic amines, gut–brain dialogue, and curing depression.

It is also well recognized that inflammation and weakened immunity are also key factors in the pathogenesis of depression (Chen et al., 2022). A line of evidence demonstrated that a significant proportion of patients with emotional-related diseases exhibit characteristics of chronic low-grade inflammation involving increased concentrations of peripheral and central inflammatory cytokines, inflammatory mediators, and acute-phase reactants (Felger 2018). For example, the levels of TNF-α, IL-1β, IL-6, IL-8 and other inflammatory cytokines in the serum of patients with depression is upregulated (Fan et al., 2017). Moreover, the study indicated that chronic methamphetamines (METH) induced anxiety-like behavior in rats, at least in part, by activating the immune Toll-like receptor 4 (TLR4) signaling pathway, which leads to the upregulation of the expression of several inflammatory agents, such as NF-κB and α-actin in the hippocampus (Wang et al., 2019; Rezaeian et al., 2020). Coincidentally, activation of NF-κB by proinflammatory mediators creates a positive feedback loop that amplifies inflammatory signals (Azam et al., 2019). Based on the above research results, Rezaeian et al. suggested that oral berberine (100 mg/kg) modulated neuroinflammation and reduced anxiety behavior via suppressing the activation of TLR4 and NF-κB in METH-addicted rats (Rezaeian et al., 2020).

### The effect of Chinese medicine prescription of berberine on depression

The diversity of bioactive compounds contributes to prescriptions of traditional herbal medicines to perform multiple pharmacological effects. From this perspective, a simple theoretical conjecture is that herbal formula with several phytochemical components have more action targets than a single-compound Chinese medicine preparation. Although the action pathway or mechanism of Chinese medicine prescriptions with an antidepressant effect is relatively obscure owing to their multi-active ingredients or complexes and non-additivity, we cannot ignore the potential of prescriptions in antidepressants. Huang-Lian Jie-Du Decoction (HLJDD) is an ancient Chinese medicine prescription composed of *Coptis chinensis* Franch, *Scutellaria baicalensis* Georgi, *Phellodendron chinense* Schneid, and *Gardenia fructus* Ellis, which can ameliorate multiple central nervous system diseases, such as cerebral ischemia (Wang et al., 2013), Alzheimer’s disease (Liu et al., 2019), and mental disorders (Qu et al., 2021). A study reported that the protective mechanism of HLJDD against neurotoxicity may be due to the protective effect of berberine, an important component of the formula, in alleviating the decrease in paraquat-induced mitochondrial membrane potential in human SH-SY5Y cells (Lee et al., 2021). In addition, previous studies on pharmacological properties of berberine have shown that it could reduce NADPH oxidase activity, attenuate mitochondrial defects and redox imbalance, and induce mitochondrial biogenesis, which may partially support the viewpoints mentioned above. The network pharmacology and metabolomics study showed that the main target of HLJDD-ameliorated depressive-like behaviors in CUMS mice was tryptophan metabolism, in which sodium-dependent serotonin transporter (SLC6A4) and monoamine oxidase A (MAOA) were regulated by active ingredients such as berberine (Qu et al., 2021).

## Challenges and possible solutions

In normal conditions, oral delivery and parenteral administration are relatively ideal routes of administration in treating chronic diseases. Although berberine has been researched for many years and received positive therapeutic feedback in an animal model of depression, there is poor bioavailability of oral or parenteral administration due to low absorption and intestinal first-pass effect, restricting its utilization in the treatment of depression (Fan et al., 2019). Therefore, it is necessary to overcome the problem of bioavailability and improve its therapeutic effects before it can be applied to clinical treatment. Nanotechnology-based drug delivery strategies constitute a potential approach to enhance the poor bioavailability of berberine. Many studies have demonstrated that topical delivery has the characteristics of targeting and performance release controllability, which plays an important role in medical treatment (Dare et al., 2020). Among them, intranasal delivery, as a form of topical delivery, bypasses the blood–brain barrier through the olfactory pathway and trigeminal nerve pathway, directly transporting drugs to the brain (Dhuria et al., 2010). Recent studies have shown that intranasal delivery of berberine was effective in the treatment of depression through a pre-prepared self-assembled thermosensitive *in situ* hydrogel (Wang et al., 2020; Xu et al., 2021). It is noteworthy that intranasal administration of hydrogel increased the bioavailability of berberine by approximately 135-fold, compared with intragastric administration of drug solution, which is probably the positive result of the synergistic effect of hydrogels in drug delivery.

## Discussion

Depression is essentially a disease of the brain. Once its mechanisms of action with antidepressants are fully known, this disorder is likely to be effectively prevented and treated through some targets. In actual clinical treatment, antidepressants, such as selective serotonin reuptake inhibitor (SSRIs) or serotonin–noradrenaline reuptake inhibitors (SNRIs), are often applied to relieve symptoms. Actually, studies demonstrated that up to more than half of depression patients treated with SSRIs or SNRIs showed multiple common and well-documented adverse effects, such as some degree of emotional blunting (Christensen et al., 2021). For these reasons, while developing multitarget chemicals to reduce adverse reactions and long-term intolerance and improve the efficacy of depression drugs, a whole lot of attention is paid to the positive effects of active ingredients in natural products on depression. As a promising medicine ingredient candidate for the treatment of emotional disorders, berberine needs to be studied in substantive and long-term clinical practice with the help of neuroscience, genomics, and technologies. It exerts antidepressant effects through regulating monoamines, alleviating the dysfunction of nerve injury, ameliorating the dysregulation of immune and inflammation, and other regulatory mechanisms. Besides, it can also relieve depression-related complications, such as insomnia and cognitive impairment. Therefore, programs are necessary to increase knowledge on the use of natural antidepressants, formulate a treatment strategy for individual needs to reduce the adverse reactions of patients, as well as enhance the public’s confidence in traditional Chinese medicine to cure emotional diseases, so as to improve the public’s lack of trust in the health care system.

## Conclusion

We have reviewed the latest research progress of berberine in the treatment of depression, which may provide a reference for the search of natural antidepressants. Although berberine has been revealed with multiple therapeutic actions, and it may be a potential drug for mood disorders, transformation research is needed to further address its safety concerns, poor bioavailability, and solubility.
